# Shared Dosimetry Error in Epidemiological Dose-Response Analyses

**DOI:** 10.1371/journal.pone.0119418

**Published:** 2015-03-23

**Authors:** Daniel O. Stram, Dale L. Preston, Mikhail Sokolnikov, Bruce Napier, Kenneth J. Kopecky, John Boice, Harold Beck, John Till, Andre Bouville

**Affiliations:** 1 Department of Preventive Medicine, University of Southern California, Los Angeles, California, United States of America; 2 Hirosoft International, Eureka, California, United States of America; 3 Southern Urals Biophysics Institute, Ozersk, Russia; 4 Pacific Northwest National Laboratory, Richland, Washington, United States of America; 5 Fred Hutchinson Cancer Center, Seattle, Washington, United States of America; 6 Vanderbilt University, Nashville, Tennessee, United States of America; 7 U.S. Department of Energy, New York, New York, United States of America; 8 Risk Assessment Corporation, Neeses, South Carolina, United States of America; 9 Radiation Epidemiology Branch, National Cancer Institute, Rockville, Maryland, United States of America; Leibniz Institute for Prevention Research and Epidemiology (BIPS), GERMANY

## Abstract

Radiation dose reconstruction systems for large-scale epidemiological studies are sophisticated both in providing estimates of dose and in representing dosimetry uncertainty. For example, a computer program was used by the Hanford Thyroid Disease Study to provide 100 realizations of possible dose to study participants. The variation in realizations reflected the range of possible dose for each cohort member consistent with the data on dose determinates in the cohort. Another example is the Mayak Worker Dosimetry System 2013 which estimates both external and internal exposures and provides multiple realizations of "possible" dose history to workers given dose determinants. This paper takes up the problem of dealing with complex dosimetry systems that provide multiple realizations of dose in an epidemiologic analysis. In this paper we derive expected scores and the information matrix for a model used widely in radiation epidemiology, namely the linear excess relative risk (ERR) model that allows for a linear dose response (risk in relation to radiation) and distinguishes between modifiers of background rates and of the excess risk due to exposure. We show that treating the mean dose for each individual (calculated by averaging over the realizations) as if it was true dose (ignoring both shared and unshared dosimetry errors) gives asymptotically unbiased estimates (i.e. the score has expectation zero) and valid tests of the null hypothesis that the ERR slope β is zero. Although the score is unbiased the information matrix (and hence the standard errors of the estimate of β) is biased for β≠0 when ignoring errors in dose estimates, and we show how to adjust the information matrix to remove this bias, using the multiple realizations of dose. The use of these methods in the context of several studies including, the Mayak Worker Cohort, and the U.S. Atomic Veterans Study, is discussed.

## Introduction

Assessment of radiation exposure in many epidemiologic studies of disease is subject to considerable uncertainties. When estimation of radiation exposure is based on historical reconstructions many determinates of dose may be uncertain and affect a large number of study participants simultaneously. An important example is the Hanford Thyroid Disease study [1,2,3] which utilized the CIDER (Calculation of Individual Doses from Environmental Radionuclides) dosimetry system to estimate, approximately four decades after exposures began, individual thyroid doses due to ^131^I releases for members of the population living proximal and downwind of the Hanford site in the late 1940s and early 1950s. Uncertainties in a number of parameters including source term, atmospheric transport and deposition, biological parameters of iodine transfer into cows and goats milk, and parameters of milk production and distribution are propagated in such a way as to affect potential doses for many or all study participants simultaneously. The CIDER system was designed to represent uncertainty (both shared and unshared) by means of repeated realizations of dose based on a Monte Carlo calculation in which uncertain parameters were given uncertainty distributions and draws from those distributions were used to develop dose estimates for the entire cohort simultaneously.

In a more recent example the Improved Thyroid Dose Reconstruction System, TD-10 [4] provides thyroid doses for use in a cohort of children and adolescents in the Ukraine [5] exposed to Chernobyl radiation. The dosimetry system incorporates direct measurements of thyroid activity, and local ^131^I deposition and also the influence of dietary and lifestyle habits collected by interview as well as estimated thyroid volume and mass according to age and other factors. Similarly with the CIDER system for Hanford, the TD-10 system represents uncertainty in thyroid dose by providing multiple realizations of potential dose, in this case 1,000 cohort realizations. Little et al. [6] have described possible approaches to statistical analysis of these data including regression calibration and Monte Carlo techniques. Other examples include the methods of Puncher and Birchall [7], using the IMBA program (Public Health England) for internal dose and uncertainty estimation.

A natural question arises about how to take into account shared uncertainties either represented in this way (as many realizations) or in summary form (e.g. as a covariance matrix described below) into epidemiological analysis. In this paper we explore this question specifically in relation to two different cohorts: the Atomic Veterans Study (AVS) [8] and the Mayak Worker Cohort (MWC) [9], generalizing previous work [10] on this problem which was focused on the Hanford Thyroid Disease Study. We develop some novel mathematical expressions for the influence of shared and unshared dosimetry errors on dose-response parameter estimation that are very useful in the analysis of important special cases. Using these expressions we carry out some formal calculations for a study design question (the modification of power calculations to allow for shared dosimetry error) based on the AVS. Finally we indicate how to approach the problem of shared dosimetric uncertainty when, as in the MWC exposures (1) are prolonged over extended time periods and (2) analysis of cancer risk is based on linear excess relative risk (ERR) models.

## Effects of Measurement Error

The term measurement error is extremely broad and can refer to a huge range of issues which vary in importance and degree of difficulty, with some problems being relatively simple and straight forward, with the errors having predictable effects which are easy to correct for while other problems are nearly or completely intractable. The discussion below focuses on the effects of measurement error on parameter estimation and on correction techniques that can be applied in many epidemiologic analyses to mitigate these effects. Both independent random and non-independent, i.e. shared, errors are considered and a basic statistical framework that can be used to address uncertainty information that is embodied in complex dosimetry systems is discussed.

Errors in measurement can have many effects upon the results of epidemiologic or statistical analyses. For example Thomas et al. [11] in a review for the Annals of Public Health described effects on

(1) The power of statistical analyses to detect significant associations between an outcome Y and an exposure X, when true X is not available, but only a measured or imputed exposure, Z
(2) the shape of the dose response relationship E(Y|Z) compared to E(Y|X) where Y is the outcome of interest, X is the true exposure of interest, and Z is the measured exposure, often, but not always, E(Y|Z) will be attenuated compared to E(Y|X) so that slope parameters *β* in E(Y|X) that govern the true dose response relationship will be underestimated;
(3) the variance structure V(Y|Z) compared to V(Y|X), which can distort inferences about dose response parameters *β* even if the estimates of *β* using Z are unbiased
(4) the covariance structure, Cov(Y_1_, Y_2_|X) between two dose-dependent outcomes conditional on true dose X. For example Y_1_, and Y_2_ may be independent given X but have positive covariance Cov(Y_1_, Y_2_|Z) given measured dose. The phenomenon is known as residual confounding [12] and has been extensively discussed in the LSS [13–16].

Much of the exposure measurement error literature distinguishes (a) differential vs. non-differential, (b) random vs. systematic, (c) Berkson vs. classical, and (d) shared vs. unshared errors. Approaches to assessing and correcting for measurement error effects on statistical analyses can be classified into two broad groups as either based on a *functional* or *structural* interpretation of the error problem [17]. The distinction between the two is that functional (sometimes called non-parametric) methods make no assumptions about the form of the distribution of true dose X while structural methods include the modeling of this distribution as part of the measurement error correction problem. While the functional approach is by design robust to modeling mistakes concerning the distribution of X in a study, this robustness can come at a price, e.g. less power and flexibility than achieved by the structural approach when the distributional form of X is reasonably well known or can be inferred [18]. This review focuses upon non-differential errors and the structural approach to assessing and correcting for the effects of measurement error. Here non-differential means that the error in the exposure of interest is independent of the outcome, Y, if X is known, or equivalently that f(Y|X, Z) = f(*Y*|X) where f(Y|•) refers to the conditional probability distribution of Y given the information • on the RHS of the |. This is also expressed by saying that Z is a *surrogate* for X. Generally correcting for the effects of differential error is a less tractable problem in that all the standard approaches based on assuming non-differential errors, will tend to fail.

## Classical and Berkson Error

The terms *Classical* and *Berkson* error [19] refer to random error models that have different attributes, in a classical error model it is assumed that the estimate is independently distributed around X in such a way that E(Z|X) = X, whereas a Berkson error model assumes that X is distributed around Z with E(X|Z) = 0. We see (in the section on regression substitution below) that under a Berkson error measurement error model if we are fitting a linear response model to Y and substitute Z for unknown X the resulting parameter estimates will give an unbiased estimate of the linear dose response. The classical error model on the other hand produces attenuated estimates of the slope parameter. (Both types of error result in loss of study power).

Classical error models are often used as a representation of how an idealized (but error prone) exposure or dose meter should perform, i.e. the estimates Z should be distributed around true dose X with independent errors. Such an idealized exposure meter would be applicable to any study with any distribution of X, and the estimate Z for one individual would not depend in any way upon the distribution of other study members Z.

Berkson error models on the other hand are often used as an idealized model for averaging error. For example consider a series of inhabited islands contaminated after a nuclear accident, suppose that true X is measured at enough points and times on each island so that the exposure average is well understood. Individual doses however depend on the actual location and/or behavior of an exposed person so that the individual doses may ideally be considered to cluster around the island means. If the island means are used in the analysis then the errors in applying these to the individual inhabitants yield Berkson errors which will be independent of each other if the island means are known perfectly.

## Correcting for Exposure Measurement Error using Regression Substitution

Regression substitution [17,20,21] remains the most widely used approach to measurement error adjustment of risk estimates in epidemiologic analysis. In simplest form the method replaces a single unknown true dose X with E(X|Z) for each individual and treat these as equivalent in the regression models. The rationale for this is fairly straight-forward. If we assume a linear relationship between the expected value of outcome Y given true dose X, i.e.

E(Y|X)=α+βX (1)

then using the rule of conditional expectations the expected value of Y given measurement Z is equal to

$$\begin{array}{l} E(Y|Z)=E_{X|Z}\{E(Y|X,Z)\}\\ =E_{X|Z}\{E(Y|X)\}\text{ (this follows since Z is a surrogate for X)}\\ =E_{X|Z}(\alpha +\beta X)\\ =\alpha +\beta E(X|Z) \end{array}$$

if we denote E(X|Z) as Z* then E(Y|Z*) has the same linear slope term as does E(Y|X). This implies that we can fit the dose response model (1) by using Z* as the explanatory variable, and also implies that the presence of unshared Berkson error does not by itself bias the dose response parameter estimates. The regression substitution approach can be extended to include other covariates (i.e. adjustment variables) and often works well even when mildly non-linear models are being fit, such as logistic or Cox regressions when effect estimates are not extremely strong [21]. We note that (1) can be generalized to include additional covariates and interactions. Other methods for correcting for measurement error have been considered of course, these include the SIMEX [22] method and structural equation modeling [11], among others. We do not consider these further here since it seems difficult to extend these methods to deal with, as described next, dosimetry systems that provide multiple realizations of dose.

## Shared Error and Complex Dosimetry Systems for Dose Reconstruction

### Exposure reconstruction

In many settings no actual "measurements" exist in the literal sense. Instead knowledge of the physical processes that produced dose and that transfer radionuclides (in the case of radiation exposure in the environment) from source points to populations is used to reconstruct exposures to radiation that may have taken place many years ago. While such dose reconstruction is not new, there recently has been an emphasis on explicitly including uncertainty estimation as part of the best estimation of individual doses. Starting with thyroid and leukemia disease studies in Utah in the 1990s [23,24] and notably for ^131^I exposures originating from the Hanford facility [25–28] Monte Carlo calculations have formed the basis for both the calculations of estimated dose as well as the representation of uncertainty in estimated dose. We first describe the Hanford study and many of the points made by Stram and Kopecky [10] about the proper incorporation of these Monte Carlo systems into dose response estimation.

### Complex dosimetry systems

Using the terminology of Stram and Kopecky [10], a complex dosimetry system is one in which multiple possible doses (dose realizations) are computed for all members of the cohort with variability in the possible doses representing dosimetric uncertainty. In complex dosimetry systems shared uncertainty is to be reflected in the way that dose realizations co-vary from individual to individual. If two individuals share an important uncertainty about a factor determining dose then their dose estimates will tend to be highly correlated over the replications, if they do not, then they will tend to be almost independent.

Underlying Stram and Kopecky's approach is the assumption that a complex dosimetry system provides realizations that can be regarded as being sampled from the distribution of true dose given all that is known, **W**, about the determinates of true dose.

To give a concrete example consider the effects of uncertainty in the size of the source term (total ^131^I released) for the Hanford Study. The degree of uncertainty is finite, because much is known about the history of the Hanford nuclear site, but is not zero either. Crudely speaking the Monte-Carlo method generates, for each replication of cohort dose, a realization of source term size from a prior distribution of possible values of this source term given all that is known about this parameter, and then uses this value in all subsequent calculations for that replication; thus error (the difference between true and simulated) in the source term affects all doses simultaneously. In other steps of the calculation for the same replication there may be sharing that is not so extensive; for example drinkers of cow's milk will share certain additional uncertainties in their total dose, not shared with non-milk drinkers.

In addition to these shared or partly shared sources of uncertainty there is the possibility that input data (location during exposure times, milk drinking habits, etc.) that has been elicited for each individual may be subject to independent—generally classical error. Note that building the dosimetry system may require some statistical analysis of the input data before the using the Monte Carlo methods all the way down to calculate individual dose. For example if milk consumption for participants in the Hanford study is reported with independent classical error, the selection of a random milk intake by the dosimetry system should be from the conditional distribution of true intake given reported intake. Specifically, if R is the reported intake and D is the true amount consumed a classical error model may be used to represent the relationship between R and D. Then the conditional distribution is

$$f(D|R)=\frac{f(R|D)f(D)}{\int f(R|D)f(D)dD}$$

and Monte Carlo sampling should be from f(D|R). This integration requires knowledge of *f*(*D*) and f(R|D) which can be imperfect itself. Generally if one can specify f(R|D) then it is possible to approximate *f*(*D*) from the observed distribution of *R* by using deconvolution methods such as those described in Pierce and Kellerer [18] so that the key issue is knowledge of the form and parameters of *f*(*R*|*D*); uncertainties about f(R|D) could also be incorporated into the Monte Carlo calculations if an appropriate prior distribution can be agreed upon.

### Incorporating multiple realizations of dose into dose-response analysis

If we accept the notion that the Monte Carlo-based complex dosimetry system provides samples of cohort doses given everything that is known about true dose, i.e. we treat each cohort dose replication, X^r^, as sampled from a probability distribution f(X|**W)** of true dose X for the entire cohort given the state of knowledge, **W**, about determinants of true dose for each individual, then we can consider several possible methods for estimating parameters in a risk or hazard model for Y given X. Here X indicates the N-dimensional vector of true doses for all N individuals, and X^r^ is the N-vector of realized dose for replication number *r*. We can turn the entire problem into a Berkson error problem by obtaining enough realizations X^r^ so that the sample average of these (averaged over r) is equal to the mean of true dose given **W**. The first step in the analysis of Stram and Kopecky [10] is to use this average dose as a replacement for true dose in the dose response model f(Y|X) in order to provide initial estimates of the dose response parameters β of primary interest. Note that these initial estimates may be unbiased or close to unbiased (using the arguments above) but nevertheless the standard error estimates may flawed either because the variance function, Var(Y|Z), changes compared to Var(Y|X) or because of loss of independence between the individual outcomes due to shared error.

Stram and Kopecky then go on to propose some rather simple ad hoc methods for determining the impact of shared errors on the standard errors of the parameter estimates and on testing for non-zero effects of exposure. In addition they make some general points about the impact of shared errors.

1. Ignoring shared error in the dosimetry system does not affect the asymptotic size of the test of the null hypothesis that there is no dose-response relationship between exposure and outcome (i.e. the test that β = 0 remains valid)
2. However, sample sizes or the power of a test of the null hypothesis calculated under a specific alternative hypothesis (β ≠ 0) will be incorrect if they ignore shared errors; i.e. for a given sample size power will be over-estimated.
3. Confidence intervals will also be affected. Ignoring shared dosimetry error while constructing confidence intervals will result in confidence intervals that are too narrow.
4. However, because of point 1, a naïve confidence interval for β ignoring dosimetry error that does not overlap 0 will not overlap 0 once shared errors have been accounted for. In this sense it is often the upper confidence bounds for (positive) β that are most affected by shared dosimetry error.

The ad hoc correction techniques described in Stram and Kopecky [10] were based upon decompositions of the covariance matrix of true dose X given **W** into a single shared additive, single shared multiplicative, unshared additive and unshared multiplicative components. In the following we also base our analysis upon the covariance matrix of true dose X given **W** but we take a more rigorous approach (allowing for a more general covariance structure).

Note that since the dosimetry system gives a large number of dose replications we can approximate Var(X|**W**) as

$$\frac{1}{L}\sum_{r=1}^{L}\left(X^{r}-Z\right)(X^{r}-Z)\prime$$

where L is the number of replications drawn from f(X|W) and Z is the sample mean of X^1^, …, X^L^.

We now consider simple linear models basically evaluating a "plug in" version of the score equation. We assume initially that the outcome data Y = (*Y*_*1*_, *Y*_*2*,_ … *Y*_*N*_) conditional on true value of exposure X = (*X*_*1*_, *X*_*2*_, …, *X*_*N*_) is a vector of independent Poisson random variables with mean E(*Y*_*i*_) = α_0_+*β X*_*i*,_ or else a vector of independent Gaussian random variables also with mean E(*Y*_*i*_) = α _0_+*β X*_*i*,_ and known residual variance σ^2^. Let *θ* denote the vector of parameters to be estimated, e.g. here *θ* equals(α _*0*_,*β*)ʹ. Using the average dose, *Z*, over the replications we fit the model E(*Y*_*i*_) = α _0_+*β Z*_*i*,_ (in matrix notation*E*(*Y*) = **Z** *θ*where the bolded **Z** denotes a Nx2 matrix with the first column equal to a vector of 1s and the second column equal to the average dose *Z*)

The second step is to adjust the variance of the estimate of *θ*, denoted $\hat{\theta }$, by using the Fisher's scoring approximation[29]

$$\hat{\theta }-\theta \approx I_{w}{}^{-1}S_{w}$$ (2)

where *I*_w_is the expected information matrix (i.e. minus the expected value of the second derivative of the log likelihood), here a 2 x 2 symmetric matrix with diagonal and off diagonal terms being the sums $\sum_{i=1}^{N}\frac{1}{Var*(Y_{i}|Z_{i})}$, $\sum_{i=1}^{N}\frac{Z_{i}{}^{2}}{Var*(Y_{i}|Z_{i})}$ and $\sum_{i=1}^{N}\frac{Z_{i}}{Var*(Y_{i}|Z_{i})}$ respectively, and where *S*_*w*_ is the naïve score vector (first derivative of the log likelihood), with terms $\sum \frac{1}{Var*(Y_{i}|Z_{i})}[Y_{i}-(\alpha_{0}+\beta Z_{i})]$ and $\sum \frac{1}{Var*(Y_{i}|Z_{i})}[Y_{i}-(\alpha_{0}+\beta Z_{i})]Z_{i}$. Here *Var* * (*Y*_*i*_ | *Z*)is the naïve variance of *Y*_*i*_ ignoring the errors in using Z to estimate X. Specifically for the Poisson model *Var* * (*Y*_*i*_ | *Z*_*i*_)equals the mean, here *α*_*0*_ *+β Z*_*i*_, and for the Gaussian equals the true residual error σ^2^ (we relax this below).

If there are no dosimetry errors then from basic likelihood theory we have that *Var*(*S*_*w*_) = *I*_*w*_ so that from (2),$Var(\hat{\theta })=I_{w}^{-1}Var(S_{w})I_{w}^{-1}=I_{w}^{-1}$. If there are dosimetry errors but *E*(*Y*) = *a*_0_ + *βX* and *E*(*X* | **W**) = *Z* then we see immediately that the plug in score equation is unbiased so that solving the score equation (finding the parameter estimates that zero the scores) should give asymptotically valid parameter estimates. The naïve variance estimate of $\hat{\theta }$ (i.e.$I_{w}{}^{-1}$) however is biased. The true variance is $Var(\hat{\theta })=I_{w}{}^{-1}Var(S_{w})I_{w}^{-1}$, and*Var*(*S*_*w*_)can be evaluated as

$$E_{X|W}\{Var(S_{W}|X)\}+Var_{X|W}\{E(S_{w}|X)\}$$ (3)

We note for the Poisson model a little algebra shows that since *Var*(*Y*_*i*_) = *α*_0_ + *βX*_*i*_ *E*_*X\W*_{*Var*(*S*_*w*_ | *X*)} turns out to be just *I*_*w*_. Further *E*(*S*_*w*_ | *X*) is just a linear function of X and so we can easily calculate it in repeated draws from the complex dosimetry system. Specifically *E*(*S*_*w*_ | *X*)can be written as *c* + *β* **M**ʹ*X* where c is constant vector (not a function of X) and **M** is a N x 2 matrix with each row equal to ($\frac{1}{\alpha_{0}+\beta Z_{i}}$,$\frac{Z_{i}}{\alpha_{0}+\beta Z_{i}}$) so that the total variance of $\hat{\theta }$ will be equal to

$$I_{w}^{-1}+\beta^{2}I_{w}^{-1}M\prime Var(X|W)MI_{w}^{-1}$$ (4)

From this we can correct the standard errors of $\hat{\theta }$ from the "naïve" regression using this function taking account of all sources of shared and unshared of uncertainty. For the Gaussian linear model a similar calculation shows that *Var*(*S*_*w*_) is equal to

$$\frac{1}{\sigma^{2}}Z\prime Z+\frac{\beta^{2}}{\sigma^{4}}Z\prime Var(X|W)Z$$

where **Z** is (again) a matrix with a vector of 1's as its first column and (*Z*_1_,*Z*_2_, …, *Z*_n_)ʹas its second column.

Therefore the total variance of $\hat{\theta }$ will be equal to

$$\sigma^{2}{\left(Z\prime Z\right)}^{-1}\left[\frac{1}{\sigma^{2}}Z\prime Z+\frac{\beta^{2}}{\sigma^{4}}Z\prime Var(X|W)Z\right]\sigma^{2}{\left(Z\prime Z\right)}^{-1}$$

which simplifies to

$$\sigma^{2}{\left(Z\prime Z\right)}^{-1}+\beta^{2}{\left(Z\prime Z\right)}^{-1}Z\prime Var(X|W)Z{\left(Z\prime Z\right)}^{-1}$$ (5)

which is of form (4) above with **M** = **Z** / σ^2^.

Note that many of the properties 1–4 above (from Stram and Kopecky) can be seen to apply to equation (6) and equation (7). For example if we are interested in testing the null hypothesis that *β* = 0 then we can drop the second term (since it is 0 under the null) in (4) and therefore rely on the "usual" score test ignoring errors in dosimetry altogether. It is only in evaluating the variance of $\hat{\theta }$ away from the null hypothesis that the second term "kicks in" and eventually dominates the calculations as *β* ^*2*^ increases.

We can extend this to include (non-dose-dependent) covariates **U** readily. If necessary we can include X^2^ in the model, i.e. if fitting a linear quadratic dose response is important, this can be readily accomplished although we will then need to calculate each of Var(X|W), Var(X^2^|W) and Cov(X,X^2^|W) (each of these is a N x N matrix) in order to calculate standard errors that take into account shared dosimetry error

### Examining covariances at study design; an example using the Atomic Veterans Study

Implications of the above analyses are that the effect of shared Berkson error on inference depends on both the likely strength of the dose response and the extent of sharing. Preliminary studies may indicate that efforts to develop a full-fledged complex dosimetry system will only provide marginally useful information for a particular study, if sharing is deemed to be relatively small. For example if the structure of the problem is that most of the matrix Var(*X*|**W**) is equal to zero, i.e. most pairs of individuals do not share dosimetry errors, then the effect on standard errors of dose-response estimates is likely to be very small, and a full Monte Carlo-based complex dosimetry system may be only marginally more useful than a calculation simply aimed at providing simply a single "best" approximation to E(*X*|**W**). More importantly perhaps is that significant resources can be expended without improving the results of the analysis.

We use as an illustrative example the military personnel participating in nuclear weapons testing at the Nevada Test Site and the Pacific Proving Grounds from 1946 through 1962 [8]. For the testing of nuclear devices at the Bikini Atolls most of the veterans at the Pacific tests were onboard ships or on islands where there was some exposure information collected to estimate dose. If the average dose onboard each ship and on each island is well known then purely Berkson unshared error would result meaning that standard statistical methods (perhaps with a correction for over dispersion of counted data) could be utilized with little fear that epidemiological analysis will be adversely affected. Even if average doses onboard ships or on islands were poorly estimated exposure errors were generally only shared between individuals on the same ship or same island during a given test, with no sharing between ships or between tests, implying that "most" of the off diagonal terms of Var(X|W) are zero. In this case the effect of dose errors on dose response inference and estimation may also be quite limited.

In order to give some sense of what is likely for the Atomic Veterans Study, we consider the following experiment using example doses drawn from members of the AVS cohort [8]. Because doses in the AVS project are still being estimated and are not final, in this example we use doses to the 1,782 atomic veterans that were developed for compensation purposes and were known to be “high sided” (i.e. taking upper bounds when uncertain) as required by law. About 8% of the 1,782 doses are estimated to have non-zero (positive) correlations between individual’s dose, primarily because of being shipmates, participating in the same military maneuver, observing the same test, or being exposed to fallout from the same test on the same Pacific island. Also for illustrative purposes we make two additional assumptions (also "high sided" in terms of its effect on estimation): first that each dose estimate has a coefficient of variation of 50 percent so that the variance of the dose estimate is equal to 0.25 times the square of the dose estimate, and also that all non-zero correlations are in fact equal to 1. The data used in this example are available in the supplementary material provided (S1 Dataset and S2 Dataset).

Under these assumptions we consider the effect on confidence limits of an analysis of a continuous outcome (1) assuming no errors in doses, and (2) accounting for the error structure, i.e. Var(X|Z), of the dosimetry as described above. In particular if Z is the "high-sided" dose and C is the correlation matrix described immediately above then Var(X|W) is equal to $C•\left[\frac{1}{4}ZZ\prime \right]$ where •is element-wise matrix multiplication.

**Fig 1:**
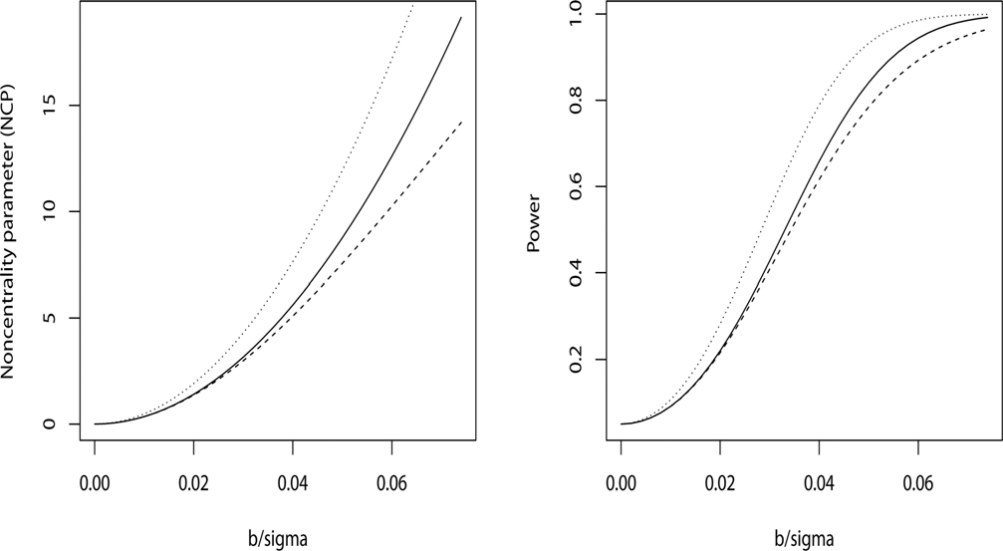
Noncentrality and Power. Points indicate effects assuming no sharing of errors, dashes include the shared error effects. For reference the dotted lines show noncentrality parameters and power assuming that true dose rather than estimated dose was available for the study. Results are particular to the AVS data described herein.

Fig. 1 shows the effect of measurement error on the noncentrality parameter (NCP), defined as $\beta^{2}/Var(\hat{\beta })$, for the test of the null hypothesis, and on the power to detect a nonzero effect of exposure assuming models

$$Y_{i}=\alpha_{0}+X_{i}\beta +e_{i}\text{ with Var}(e_{i})=\sigma^{2}$$

and

X=Z+w

with *w* having mean 0 and variance equal to Var(*X*|*Z*) described above. In each plot three lines are shown. The lowest dashed line gives the noncentrality parameter and power for testing for a nonzero slope parameter *β* in these data assuming the shared Berkson error model above, here the effect size *β* is scaled in units of*σ*. The NCP is equal to the square of slope *β* divided by the variance of the estimate of *β i*.*e*. from equation(5). The middle solid line gives the NCP and power assuming only a Berkson error model without sharing, i.e. the off diagonal values of Var(X|Z) have been set to zero but the diagonal values kept the same. For comparison purposes the upper dotted line depicts the NCP and power which would be available if there were no dosimetry errors at all, i.e. if X were equal to Z. Notice that in Fig. 1b that in this example much of the lost power due to dosimetry error has nothing to do with sharing of errors, i.e. the solid line (unshared Berkson errors) is closer to the lower dashed line (shared Berkson errors) than the upper dotted line (no dosimetry errors).

### Effects of shared errors on confidence interval coverage

In the unshared Berkson model the usual ordinary least squares (OLS) estimate of linear regression parameters *α* and *β* are unbiased although power and accuracy of the estimates is reduced (as shown above) relative to there being no dosimetry errors. Because the model above includes inhomogeneous errors (larger doses have larger errors) the OLS estimates while remaining unbiased, have a variance that is not fully captured by the usual OLS standard errors when *β* ^*2*^ is greater than zero even if there is no sharing, and sharing of error further compounds this problem. In order to directly address the simultaneous effect of inhomogeneous and shared errors on confidence interval coverage in the current example, we have to continue the analysis.

For notational convenience below we write Var(*X* | **W**) as equal to $\sigma_{X|W}^{2}K$ with the constraint that tr(**K**) = N in order to uniquely specify $\sigma_{X|W}^{2}$. (Here the trace, tr (**K**), is the sum of the diagonal elements of **K** and N is the number of study subjects, i.e. N = 1782). Now consider what happens when the matrix **K** (which specifies the dose error sharing) is ignored and OLS regression is performed. The (now inappropriate) estimate of the variance of ${\hat{\theta }}_{OLS}$ is ${\hat{\sigma }}_{OLS}^{2}{\left(Z\prime Z\right)}^{-1}$, where ${\hat{\sigma }}_{OLS}^{2}$ is the usual estimate (sum of squares of the residuals divided by n-2). Since we can write the estimate of ${\hat{\sigma }}_{OLS}^{2}$ as $\frac{1}{n-2}Y\prime (I-P)Y$ with **P** = **Z**(**Z**ʹ**Z**)^-1^ **Z**ʹwe can calculate the expected value of ${\hat{\sigma }}_{OLS}^{2}$ in the presence of shared Berkson errors as

$$\begin{array}{l} E({\hat{\sigma }}_{OLS}^{2})=E\{\frac{1}{n-2}Y\prime (I-P)Y\}=\frac{1}{n-2}\text{tr}\{(I-P)E(YY\prime )\}=\\ =\frac{1}{n-2}\text{tr}\{(I-P)[\text{Var}(Y)+E(Y)E(Y\prime )]\} \end{array}$$

(here we use the result that tr(**AB**) = tr(**BA**) for conformable matrices **A** and **B**). Note that

$$(I-P)E(Y)E(Y\prime )=Z\theta \theta \prime Z\prime -Z\theta \theta \prime Z\prime Z{\left(Z\prime Z\right)}^{-1}Z=0$$

so that the above is

$$\begin{array}{l} \frac{1}{n-2}\text{tr}\{\left(I-P\right)[\sigma^{2}I+\beta^{2}\sigma_{X|W}^{2}K]\}=\frac{\sigma^{2}}{n-2}\text{tr}\{(I-P)\}+\frac{\beta^{2}\sigma_{X|W}^{2}}{n-2}\text{tr}\{(I-P)K\}\\ =\sigma^{2}+\frac{\beta^{2}\sigma_{X|W}^{2}}{n-2}\text{tr}\{(K-PK)\}=\sigma^{2}+\frac{\beta^{2}\sigma_{X|W}^{2}}{n-2}\text{tr}\{(K-Z\;{\left(Z'Z\right)}^{-1}\;Z\prime K)\}\\ =\sigma^{2}+\frac{\beta^{2}\sigma_{X|W}^{2}}{n-2}\text{[tr}\{K\}-\text{tr\{}{\left(Z'Z\right)}^{-1}\;Z\prime KZ\}] \end{array}$$

From all this we have that the expected value of the estimator of ${\text{Var}}_{OLS}(\hat{\theta })$ (i.e. ${\hat{\sigma }}_{OLS}^{2}$ **(ZʹZ**)^-1^) is

$$\sigma^{2}{\left(Z'Z\right)}^{-1}+\frac{\beta^{2}\sigma_{X|W}^{2}}{n-2}{\left(Z'Z\right)}^{-1}\text{[tr}\{K\}-\text{tr\{}{\left(Z'Z\right)}^{-1}Z\prime KZ\}]$$ (6)

Finally subtracting (6) from (5) we get the difference between the true variance of ${\hat{\theta }}_{OLS}$(accounting for dependent outcomes Y) compared to the expected value of its estimated variance calculated assuming independence when independence doesn't hold. This difference is

$$\beta^{2}\sigma_{X|W}^{2}{\left(Z\prime Z\right)}^{-1}\left[Z\prime KZ{\left(Z\prime Z\right)}^{-1}\text{+}\frac{\text{tr}\left\{{\left(Z\prime Z\right)}^{-1}Z\prime KZ\right\}}{n-2}-\frac{\text{tr K}}{n-2}\right].$$

Now we are only really interested in the (2,2) element of this 2x2 matrix, i.e. the component that describes the difference between true and estimated variance of $\hat{\beta }$ i.e. the slope estimate only (we can term this the inflation in the variance of the slope estimate due to inhomogeneous or shared dosimetry errors). A little more straightforward but tedious algebra shows that the (2,2) element is equal to

β2σX|W2Z*′Z*[Z*′KZ*Z*'Z*(1+1n−2)−tr(K)n−2+1′K1n(n−2)] (7)

where the vector Z* is equal to $Z-\bar{Z}$ so that Z* has arithmetic mean 0. Notice that this variance inflation is zero if either β^2^ or $\sigma_{X|W}^{2}$ is zero or if **K** is the identity matrix. This last point corresponds to the well-known observation that OLS regression is unbiased and gives appropriate standard errors [30] if measurement errors are homogeneous, independent, and Berkson. As shown above power however is reduced by homogeneous Berkson measurement errors since in this case ${\hat{\sigma }}_{OLS}^{2}$ from above has expectation $\sigma^{2}+\beta^{2}\sigma_{X|W}^{2}$ and since the variance of estimated dose Z is on average smaller than is the variance of unmeasured true dose X.

Now consider the formation of confidence intervals in the presence of shared errors as in the AVS example. We use Wald test-based 95 percent confidence intervals of the form

$$\hat{\beta }\pm 1.96(\sqrt{\text{Var}(\hat{\beta })}).$$ (8)

**Fig 2:**
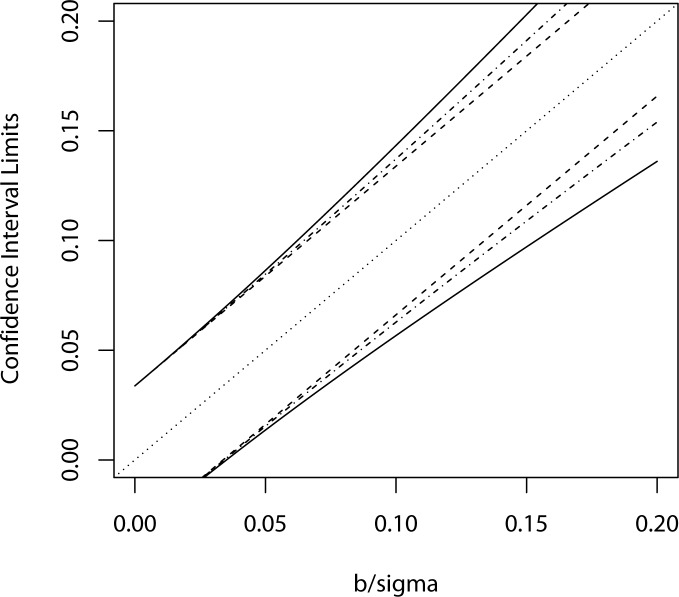
Effect of accounting for shared dosimetry errors on the length of standard errors in the high-sided calculations performed for the AVS study. The two dashed lines are based on ordinary least squares calculations and show the upper and lower bounds of a "naïve" confidence interval for slope parameter *b* (normalized by residual standard deviation, *σ*) ignoring inhomogeneous or shared errors. The solid lines show the effect of accounting for both inhomogeneous and shared error in expanding the confidence limits. The dot-dash lines between the dashed and solid lines shows the effect of adjusting for inhomogeneous errors but where there are no shared errors (off diagonals of matrix K are zero).

If we take proper account of both shared and unshared errors then the $\sqrt{\text{Var}(\hat{\beta })}$ that we use in (8) will be equal to the square root of the (2,2) element of the matrix shown in (5). However if we ignore all dosimetry errors we will in effect (at least on average) be using instead the (2,2) element of (6). Fig. 2 compares the lengths of the confidence intervals so created as a function of the true value of slope *β* in this study (again in units *σ*). Fig. 2 shows that the effect of either inhomogeneous errors or inhomogeneous error plus shared error on the ability to make inference about the slope parameter is quite modest, and is only really discernible for very strong dose response relationships (where the lower confidence intervals using all methods are very far from the null zero line).

### Complex dosimetry system for the Mayak Worker Cohort

The Mayak Worker Cohort (MWC) includes almost 26,000 men and women who first worked in one of the main plants (reactor complex, radiochemical, or plutonium production) or selected auxiliary plants (water treatment and mechanical repair) of the Mayak Production Association between 1948 and 1982. All cohort members had some potential for external gamma exposures while the 17,000 radiochemical and plutonium production workers could also be exposed to plutonium, primarily as a consequence of inhaled plutonium aerosols. Film badges were used to monitor gamma exposures. Individual film badge readings were available for more than 80% of the years worked by cohort members and in all years worked for 72% of the cohort members, about 20% of the cohort members have no badge dose data. Estimates of an individual’s annual external doses were reconstructed based on work history for years in which a badge dose record was not available.

Plutonium exposure resulted in chronic long term exposure to the lung, liver, and bone surface with very small exposures to other organs. Plutonium intakes and annual doses have been estimated for all of the radiochemical and plutonium plant workers for whom urine bioassay data were available (about 8,000 workers). These estimates are based on limited data on workplace- and time-dependent Pu aerosol concentrations together with complex and highly uncertain models for plutonium absorption and metabolism. Shared errors are a major component of the Pu dose uncertainties. No efforts have (yet) been made to reconstruct Pu dose estimates for workers without bioassay data (“unmonitored” workers) and, for the purpose of the estimation of Pu dose effects, follow-up for monitored workers begins two years after the initial monitoring.

The most recent dosimetry system for the MWC, the Mayak Workers Dosimetry System 2013 (MWDS 2013) provides Monte-Carlo realizations of dose histories for both external gamma dose and internal (alpha-particle) dose from plutonium ingestion, these are specifically designed to be interpretable as samples from the distribution of possible true dose given what is known about dose determinants. While use of these dose realizations in epidemiologic analysis has just begun it is clear that the many shared uncertainties regarding internal dose estimation are reflected in much larger variability of individual dose estimates for internal than external dose, and in much higher correlations between cohort members dose histories for internal than external dose. Therefore it seems especially important to formally incorporate the uncertainties in internal dose estimation into the epidemiological analysis of this cohort.

There are two main extensions needed in order to adapt the measurement error correction of the information matrix to the types of hazard function regression used to analyze the MWC data. The first is to an extended ERR model for Poisson regression, and the second is to the prolonged exposures and follow-up times relevant to the MWC.

### Linear excess relative risk model

Here we consider measurement error correction of a linear ERR model often used to model event time data, including cancer mortality in the MWC. Specifically we have

$$E(Y_{i})=\exp(\alpha_{0}+\alpha_{1}A_{i})(1+\beta X_{i}\exp(\alpha_{2}C_{i})).$$

Here covariates *A*_*i*_ modify background rates of disease not related to dose (such as age sex, etc.) the linear term in dose (*β* the ERR per unit dose) multiplies the baseline risk, and dose modifiers *C*_*i*_ alter the slope of the regression depending on various covariates; for example age at exposure or time since exposure can act as a modifier of excess risk but not of baseline rates. For this model we have *θ* = (α_0_,α_1_,α_2_,*β*)ʹ and the variance of $\hat{\theta }$ is of form (4) with

$$\begin{array}{l} I_{w}=\\ \sum_{i=1}^{N}\exp(\alpha_{0}+\alpha_{1}A_{i})(1+\beta Z_{i}\exp(\alpha_{2}C_{i}))\left[\begin{array}{c} 1\\ A_{i}\\ C_{i}\frac{\beta Z_{i}\exp(\alpha_{2}C_{i})}{1+\beta Z_{i}\exp(\alpha_{2}C_{i})}\\ \frac{Z_{i}\exp(\alpha_{2}C_{i})}{1+\beta Z_{i}\exp(\alpha_{2}C_{i})} \end{array}\right]\left[\begin{array}{cccc} 1 & A_{i} & C_{i}\frac{\beta Z_{i}\exp(\alpha_{2}C_{i})}{1+\beta Z_{i}\exp(\alpha_{2}C_{i})}, & \frac{Z_{i}\exp(\alpha_{2}C_{i})}{1+\beta Z_{i}\exp(\alpha_{2}C_{i})} \end{array}\right] \end{array}$$

and **M** equal to a N x 4 matrix with ith row equal to

$$\exp(\alpha_{0}+\alpha_{1}A_{i}+\alpha_{2}C_{i})\left[\begin{array}{cccc} 1, & A_{i}, & C_{i}\frac{\beta Z_{i}\exp(\alpha_{2}C_{i})}{1+\beta Z_{i}\exp(\alpha_{2}C_{i})}, & \frac{Z_{i}}{1+\beta Z\exp(\alpha_{2}C_{i})} \end{array}\right].$$

The remaining extension that is required of the above methods is to deal with prolonged exposure taking place during follow-up so that each individual accumulates exposure over an extended period while being followed for the outcome of interest, this is especially critical for doses due to internal deposition of plutonium to the lung, liver, and bones, which continue long past the end of employment. Poisson regression using ERR models forms a fundamental starting point for survival analysis for many cohort studies including the LSS [31] and MWC [9,32]. This is because of the very strong link between the likelihood analysis of censored survival data (time to event data) using a piecewise exponential model baseline hazards model, and the likelihood for Poisson regression, e.g. [33–35]. Here we model risk using model (9) but with X_i_ replaced by X_i_(t) which represents cumulative exposure to the *i*_*th*_ cohort member up to age t (or to age *t—l* with *l* representing a lag time).

While Poisson regression for event time data is usually described in terms of a highly stratified table of event counts and person years, individual contributions to each cell of the table can be disaggregated and modeled directly as Poisson sub-counts [33,34]. For example for a Mayak worker with 20 years of follow-up and accumulating dose over each year (e.g. due to internal plutonium exposure) that individual's record could be divided into 20 records each with an accumulated dose and at most one event for analysis using Poisson regression. Since the dosimetry system generates realizations of potential dose for all person-years, and since in the disaggregated data the event count for each person-year is assumed to be independent given true cumulative exposure, we can apply the correction described above at the person-year level (by computing a covariance matrix for all person-year accumulated dose from the replications provided).

Our analysis of continuous follow-up and accumulating dose contains one simplification worth noting, although its impact is likely to be vanishingly small in most real studies including the MWC and the AVS. Following the logic of Prentice 1982 [36] the expected value of true dose needed for each cell of the table (or each sub-count as above) actually depends upon the slope parameter *β*, and the amount of follow-up time that has passed. This reflects the fact that if the relationship between risk and exposure is positive (i.e. *β* > *0*) that individuals with higher true dose will be removed from follow-up by the occurrence of disease at a faster rate than those individuals with the same estimated dose but lower true dose, so that the distribution of true dose given **W** changes with follow-up time. Since this dilution process depends on the true value of the risk parameter being estimated it is impossible to "build" this feature into the dosimetry system. However, in practical terms, only if events due to exposure are very common compared to the total cohort size (requiring that both exposures and risk parameter *β* be very large) will this phenomenon rise to the level of concern.

## Discussion

This paper starts very differently than most discussions of the correction of risk estimates, by assuming that the distribution of true dose X given dose determinates **W** has been adequately characterized by means of a Monte Carlo system from which a large number of samples can be taken. By “adequately characterized”, we mean that the MC dose estimates can be realistically viewed as drawn from the distribution of true dose given all relevant knowledge, i.e., from f(X|**W**). Getting to this point may involve (as described briefly above) the solution of one or more measurement error problems applied to input data pertaining to individual behavior. We are treating the dosimetry system here as a “black box” that provides as many samples as needed from the appropriate posterior distribution, with our interest focused on how to incorporate the variability of these samples into the epidemiological analyses, we recognize however that developing a Monte Carlo system that adequately characterizes the uncertainties and does not introduce unintended biases is challenging.

Once a Monte Carlo dosimetry system is in place it is not always clear what an epidemiological analysis should consist of. Some authors (e.g. [37]) have advocated fitting separate models to each realization from the dosimetry system and then summarizing the results to form overall dose-response estimates and estimates of their uncertainty. For linear models Stram and Kopecky [10] show that that this approach is subject to biases towards the null that the method described here does not suffer from.

Our approach focuses implicitly on the problem when there are considerable shared errors involved. If errors are independent then other methods including numerical quadrature or 2^nd^ order approximations to the likelihood and error functions become highly tractable and this has been exploited for example in Fearn et al. [38] in a measurement error-corrected analysis of lung cancer and radon dose. Direct integration of the full likelihood when the independence assumption does not hold (as in the case of shared errors) is a much more difficult problem as partly described by Stram and Kopecky [10].

The approach of this paper is fundamentally a mixture between a completely Bayesian approach to estimation and inference applied to parameters in the distribution of X|W and a frequentist inference applied to the parameters governing dose response. This hybridization is already implicit in the structural approach to measurement error, where unknown doses are treated as random quantities unlike the model parameters which are regarded as fixed, and in other well-accepted statistical approaches including empirical Bayes random effects modeling [39]. We believe that a fully Bayesian analysis or a full likelihood analysis based on multiple realizations from f(X|**W**) when errors are shared is intractable for the reasons that were described in Stram and Kopecky [10], see also [40].

We are also are interested in the problem of judging at the design stage whether all the work required to develop a working complex dosimetry system is well-justified from a cost-benefit perspective. Our preliminary analysis of the AVS would tend to indicate that it is not, since a purposefully "high sided" calculation would indicate that the degree of sharing of dose errors in that study has negligible impact on the variance of a linear slope estimate obtained using ordinary least squares; in Fig. 2 the corrected and uncorrected confidence intervals only seem to begin to notably depart when the naive lower confidence intervals are as far from zero as they are from the true value, i.e. for $\hat{\beta }$/*σ* equal to about 0.80, this would (for these data) correspond to a naive p-value of 6.3x10^-5^ and a corrected p-value of around 6.7x10^-5^. Since we believe that the study as designed can only reject (with good power) the null hypothesis of no radiation effect at around the. 05 level, it seems to us immaterial that much stricter significance levels are slightly off target. While focusing on linear regression our discussion should remain highly relevant to the case control and case cohort analyses using logistic regression for reasons partly discussed below.

The situation for the MWC may be quite different essentially because of much greater sharing of errors in common dose determinants. This is especially true in the case of internal dose to lung, liver, and bone, due to plutonium exposure where dose reconstruction involves imprecisely known biological parameters governing such factors as the solubility of specific compounds and particle transport within the human body. An especially complex issue for Mayak is the protraction of dose over long periods of time (with dose continuing to accumulate beyond termination of Mayak employment for internal exposure)

The primary calculations for Gaussian or Poisson models (with linear link functions) involve the covariance matrix of doses, or histories of doses, over the set of possible replications. In a complex dosimetry system supplying lengthy dose histories for a large number of study subjects this correlation matrix may be extremely large. One of the virtues of the approach described here is that it is not necessary to compute the entire matrix in one calculation. For example for the linear Poisson regression this covariance matrix is used only in the calculation of the p x p matrix**M** *Var*(*X* | **W**)**M** ^*T*^where p is the number of model parameters which remains far less than the number of individuals, or of individual person-years. This calculation can be broken into many sub-calculations. Specifically if **M**_**i**_ (i = 1 … m) is a n_i_ x 2 sub-matrix of **M** and V_ij_ is a corresponding n_i_ x n_j_ sub-matrix of Var(X) then **M** ^**T**^ *Var*(*X* | **W**)**M** =$\sum_{i=1}^{m}\sum_{j=1}^{m}M_{i}^{T}V_{ij}M_{j}$. This calculation requires that each sub-matrix, V_ij_, of Var(X|**W**) to be computed only once and once used does not have to be retained. This simplification of the calculations make it possible to consider applying this correction to models fit to the Mayak data given the extremely protracted nature of dose.

There are limitations to the information adjustment for dose uncertainty utilized here, the most important of these has to do with the requirement that the Monte Carlo dosimetry system adequately characterizes the true doses, as defined above. This does appear to be a goal of these systems, but building them to really reflect current knowledge of complex unknown parameters can be daunting. Moreover there can be concerns that errors in individual input data are being overlooked as a source of independent "classical" error when the dosimetry system is actually being used. As noted above it may be important to "pre-convert" classical to Berkson error if input data is known only with individual independent errors.

The other obvious limitation is when non-linear dose response relationships are to be estimated. For binary data the extremely widely used logistic regression model is not directly amenable to the methods described above for two reasons, one is the non-linearity of the mean as a function of covariates, and the second is the non-linearity of the variance as a function of the mean, which complicates the variance calculation compared to the form in equation (6) for the Poisson model. If however, disease is rare then there is very little practical difference between the Poisson and binary models when applied to cohort data. Furthermore only if dose responses are quite strong will it be possible to distinguish between a linear and a logistic link function in terms of the fit of the model.

## Supporting Information

S1 Dataset (TXT)

S2 Dataset (TXT)
