# Correction: Shared Dosimetry Error in Epidemiological Dose-Response Analyses

**DOI:** 10.1371/journal.pone.0126041

**Published:** 2015-05-06

**Authors:** The PLOS ONE Staff

In the section Shared Error and Complex Dosimetry Systems for Dose Reconstruction under the subheading “Incorporating multiple realizations of dose into dose-response analysis,” the first sentence of the seventh paragraph incorrectly refers to equation 6 and equation 7. This should instead should refer to Eqs 4 and 5. The correct sentence is: Note that many of the properties 1–4 above (from Stram and Kopecky) can be seen to apply to Eq (4) and Eq (5).

The second sentence in the final paragraph of the Discussion incorrectly refers to equation 6. This should instead refer to Eq 4. The correct sentence is: For binary data the extremely widely used logistic regression model is not directly amenable to the methods described above for two reasons, one is the non-linearity of the mean as a function of covariates, and the second is the non-linearity of the variance as a function of the mean, which complicates the variance calculation compared to the form in Eq (4) for the Poisson model.

The publisher apologizes for these errors. Eqs 4 and 5 can be viewed here:

$$I_{w}^{-1}+\beta^{2}I_{w}^{-1}M\prime Var(X|W)MI_{w}^{-1}$$ (4)

$$\sigma^{2}{\left(Z\prime Z\right)}^{-1}+\beta^{2}{\left(Z\prime Z\right)}^{-1}Z\prime Var(X|W)Z{\left(Z\prime Z\right)}^{-1}$$ (5)
